# A novel stable isotope labelling assisted workflow for improved untargeted LC–HRMS based metabolomics research

**DOI:** 10.1007/s11306-013-0611-0

**Published:** 2013-12-04

**Authors:** Christoph Bueschl, Bernhard Kluger, Marc Lemmens, Gerhard Adam, Gerlinde Wiesenberger, Valentina Maschietto, Adriano Marocco, Joseph Strauss, Stephan Bödi, Gerhard G. Thallinger, Rudolf Krska, Rainer Schuhmacher

**Affiliations:** 1 Department for Agrobiotechnology (IFA-Tulln), Center for Analytical Chemistry and Institute for Biotechnology in Plant Production, University of Natural Resources and Life Sciences, Vienna (BOKU), Konrad-Lorenz-Str. 20, 3430 Tulln, Austria; 2 Department of Applied Genetics and Cell Biology, University of Natural Resources and Life Sciences, Vienna (BOKU), Konrad-Lorenz-Str. 24, 3430 Tulln, Austria; 3 Institute of Agronomy, Genetics and Field Crops, Università Cattolica del Sacro Cuore, Via Emilia Parmense 84, 29122 Piacenza, Italy; 4 Health and Environment Department, Bioresources – Fungal Genetics and Genomics, Austrian Institute of Technology (AIT), Konrad-Lorenz-Str. 24, 3430 Tulln, Austria; 5 Institute for Genomics and Bioinformatics, Graz University of Technology, Petersgasse 14, 8010 Graz, Austria; 6 Core Facility Bioinformatics, Austrian Centre for Industrial Biotechnology, Petersgasse 14, 8010 Graz, Austria

**Keywords:** ^13^C-labelling, Internal standardisation, Metabolomics, Fusarium, Wheat, Maize

## Abstract

Many untargeted LC–ESI–HRMS based metabolomics studies are still hampered by the large proportion of non-biological sample derived signals included in the generated raw data. Here, a novel, powerful stable isotope labelling (SIL)-based metabolomics workflow is presented, which facilitates global metabolome extraction, improved metabolite annotation and metabolome wide internal standardisation (IS). The general concept is exemplified with two different cultivation variants, (1) co-cultivation of the plant pathogenic fungi *Fusarium graminearum* on non-labelled and highly ^13^C enriched culture medium and (2) experimental cultivation under native conditions and use of globally U-^13^C labelled biological reference samples as exemplified with maize and wheat. Subsequent to LC–HRMS analysis of mixtures of labelled and non-labelled samples, two-dimensional data filtering of SIL specific isotopic patterns is performed to better extract truly biological derived signals together with the corresponding number of carbon atoms of each metabolite ion. Finally, feature pairs are convoluted to feature groups each representing a single metabolite. Moreover, the correction of unequal matrix effects in different sample types and the improvement of relative metabolite quantification with metabolome wide IS are demonstrated for the *F. graminearum* experiment. Data processing employing the presented workflow revealed about 300 SIL derived feature pairs corresponding to 87–135 metabolites in *F. graminearum* samples and around 800 feature pairs corresponding to roughly 350 metabolites in wheat samples. SIL assisted IS, by the use of globally U-^13^C labelled biological samples, reduced the median CV value from 7.1 to 3.6 % for technical replicates and from 15.1 to 10.8 % for biological replicates in the respective *F. graminearum* samples.

## Introduction

While full genome sequences have been determined for many organisms, it is currently still not possible to measure the complete metabolite inventory of a biological system due to methodical limitations. Complementary, sensitive and generic techniques are required to cope with the large chemical diversity and wide dynamic range of low molecular weight metabolites. Gas chromatography (GC) or liquid chromatography (LC) coupled to mass spectrometry (MS) as well as nuclear magnetic resonance (NMR) spectroscopy have emerged as key techniques in the field of metabolomics, as recently reviewed by e.g. Zhang et al. (2012), Patti et al. (2012b) and Zhou et al. (2012). The combination of LC with electrospray ionisation (ESI) high resolution mass spectrometry (HRMS) has proven to be particularly powerful as this technique enables the detection of a large number of known and unknown metabolites simultaneously and requires only small amounts of the biological sample (Hiller et al. 2011; Patti et al. 2012b).

Two different metabolomics concepts can be distinguished: targeted and untargeted approaches. In targeted approaches, a set of predefined known substances is determined, thus, absolute quantification of those metabolites, which are available as authentic reference standards, is feasible. In contrast, untargeted approaches try to find mass spectrometric features of all detectable metabolites, including those unknown or at least unidentified at the time of measurement. Therefore, the untargeted approach has the advantage of probing the entire, observable metabolic space and can obtain relative abundances of several hundreds to thousands of metabolites simultaneously (Patti et al. 2012b). For the automated data processing of such LC–HRMS derived metabolomics datasets, various workflows and software packages have been developed and are frequently used in untargeted metabolomics studies e.g. XCMS (Smith et al. 2006), MzMine (Pluskal et al. 2010), MetAlign (Lommen and Kools 2012) or Maven (Clasquin et al. 2012). These software tools have in common, that they extract as many features as possible from raw LC–HRMS derived metabolomics data sets. In this respect the term feature has been defined to be a bounded, two dimensional LC–HRMS signal consisting of a chromatographic peak (i.e. retention time) and a MS signal (*m/z* value) (Kuhl et al. 2012).

Despite the recent advances regarding both LC–HRMS instrumentation and data handling platforms, the comprehensive annotation of the metabolome of a biological sample of interest and subsequent metabolite identification still remain the major bottlenecks in untargeted metabolomics, especially for LC-ESI-HRMS based studies (Scalbert et al. 2009; Castillo et al. 2011; Patti et al. 2012b; Theodoridis et al. 2012; Dunn et al. 2013). This limitation can largely be attributed to the generic nature of the ESI process, unavoidably leading to LC-ESI-HRMS full scan chromatograms and spectra, containing a large proportion of background and chemical noise compared to the signals originating from true metabolites (Keller et al. 2008; Covey et al. 2009; Trotzmüller et al. 2011). Further challenges arise from the fact that a single metabolite leads to more than one ion species (e.g. isotopologue peaks, different adducts, in-source fragments and even more complex combinations of the previous species). In addition, many metabolites cannot completely be separated in the chromatographic dimension and therefore LC–HRMS measurements result in mass spectra, which contain signals from more than one metabolite.

Another obstacle of untargeted LC-ESI-HRMS based metabolomics is related to relative quantification of the detected metabolite ions, which is caused by so called matrix effects. The composition of the evaporated sample at any time point of the LC–HRMS measurement can significantly influence the ionization efficiency and leads to ion suppression or ion enhancement in the ESI source of the mass spectrometer (Tang and Kebarle 1993; King et al. 2000). Matrix effects can seriously affect signal intensities as well as precision and even limit the coverage of the metabolome (Vogeser and Seger 2010; Koal and Deigner 2010). They are difficult to overcome in global untargeted studies as the matrix is composed of the biological sample itself. Thus, except protein precipitation, sample purification is generally not a suitable option as this would largely discriminate many sample constituents of interest (Tulipani et al. 2013). Moreover, the availability of appropriate internal standards is often limited. The detailed and comprehensive study of matrix effects is laborious and challenging, thus only a few studies reported the systematic evaluation of matrix effects and their limitations on relative metabolite quantification in the field of LC–HRMS based metabolomics (Böttcher et al. 2007; Redestig et al. 2011; Tulipani et al. 2013).

With respect to the above mentioned limitations regarding global annotation of the metabolome and method performance evaluation, there is a great demand for both innovative approaches for the analytical measurement of biological samples with LC–HRMS as well as the development of novel, improved data processing algorithms.

Stable isotope labelling (SIL) is a technique, which is becoming increasingly used in different areas of metabolomics research and it shows the potential to conquer many of the elucidated limitations in untargeted metabolomics research. In this respect, SIL assisted experiments employ stable isotopes of elements such as carbon (^13^C), hydrogen (^2^H), oxygen (^18^O), nitrogen (^15^N) and sulphur (^34^S) (Klein and Heinzle 2012; Nakabayashi et al. 2013) respectively. However, ^13^C is used most commonly as the main labelling isotope, since carbon is part of virtually any metabolite. Non-labelled, partly labelled and highly (>98 %) ^13^C enriched (U-^13^C) metabolites show the same physico-chemical properties and therefore are not separated by chromatography, but can easily be distinguished by their mass to charge ratio (*m/z*) using an MS instrument.

The use of globally U-^13^C labelled biological samples enables to circumvent problems in untargeted metabolomics, such as metabolome annotation, generation of sum formulas of the detected metabolites and putative metabolite identification. It was demonstrated that the combination of ^13^C, ^15^N and ^34^S labelling for example can help to assign the number of atoms of the respective labelling element to a metabolite ion correctly and thereby facilitates annotation of metabolites by database search (Hegeman et al. 2007; Giavalisco et al. 2008; Cano et al. 2013). In addition to improved feature extraction and metabolite annotation, SIL experiments have also been successfully used to accomplish internal standardisation (IS) for quantification of metabolite levels and, thus correct ion suppression or MS signal fluctuations caused by matrix effects in LC-ESI-HRMS (Bennett et al. 2008; Giavalisco et al. 2009; Hegeman 2010). Moreover, IS by globally stable isotope labelled biological samples allow both detailed characterisation of the performance of the used metabolomics workflow as well as an improved relative quantification/technical precision of metabolomics data.

Despite the high potential of SIL assisted approaches and their successful application in various fields, to the best of our knowledge only a few data processing tools have been published for the automated evaluation of LC–HRMS data originating from labelled biological samples. For non-targeted GC–MS based metabolomics SIL assisted metabolomics, Hiller and colleagues published the Non-targeted Tracer Fate Detection (NTFD) algorithm (Hiller et al. 2013) to study labelled tracer compounds in the central metabolism (Hiller et al. 2010). Moreover, de Jong and Beecher (2012) have successfully implemented a sophisticated method termed IROA (Isotopic Ratio Outlier Analysis™) to automatically extract features differing between experimental conditions after parallel cultivation on native and U-^13^C-labelled nutrition sources. IROA is offered as a commercial metabolomics application and software programme. The R package mzMatch-ISO (Chokkathukalam et al. 2013) is a software tool for the annotation and relative quantification of SIL derived MS data with the aim to provide insight into metabolic fluxes of biological systems. It is designed to use metabolomics data analysed by XCMS (Tautenhahn et al. 2008) and allows an in depth evaluation and visualisation of the associated isotopic patterns and their respective abundances of various native and labelled metabolites. To the best of our knowledge, MetExtract, which has been developed in our laboratory, is to date the only publicly available tool aiming at the untargeted, automated global detection of truly metabolite derived LC–HRMS signals originating from natural (compounds showing a natural carbon isotopic distribution pattern are termed “non-labelled” in the following) and stable isotope labelled biological samples (Bueschl et al. 2012).

Here a detailed analytical and data processing workflow for SIL assisted untargeted LC–HRMS based metabolomics experiments is presented. This workflow is exemplified by two representative experiments: In the first approach the filamentous fungus *Fusarium graminearum* is cultivated in parallel on a non-labelled and a U-^13^C labelled carbon source respectively under identical conditions. In the second approach a metabolomics experiment is performed using a non-labelled carbon source for cultivation of biological samples (wheat and maize), while globally U-^13^C labelled biological reference samples are used for IS. The concept and performance of both variants are presented in detail.

## Materials and methods

### Chemicals and biological samples

Acetonitrile (ACN, HiPerSolv Chromanorm, HPLC gradient grade) was purchased from VWR (Vienna, Austria); Methanol (MeOH, LiChrosolv, LC gradient grade) was purchased from Merck (Darmstadt, Germany); formic acid (FA, MS grade) was obtained from Sigma-Aldrich (Vienna, Austria). Water was purified successively by reverse osmosis and an ELGA Purelab Ultra-AN-MK2 system (Veolia Water, Vienna, Austria). Components of the modified FMM were purchased from the following suppliers: Fluka (KH_2_PO_4_, Fe(NH_4_)_2_(SO_4_)_2_·6H_2_O), Roth (MgSO_4_·7H_2_O, KCl, ZnSO_4_, H_3_BO_3_), Sigma Aldrich (NaNO_3_, MnSO_4_, CuSO_4_·5H_2_O, Na_2_MoO_4_·2H_2_O), Serva (citric acid) and VWR (glucose). U-^13^C_6_-glucose with a ^13^C enrichment degree of 99 % was obtained from Eurisotop (Saarbrücken, Germany). U-^13^C labelled wheat ear (>97 % ^13^C, cultivar Baldus), and U-^13^C labelled maize kernels (>97 % ^13^C, cultivar Yukon chief) were obtained from Isolife (Wageningen, The Netherlands).

### Cultivation of *Fusarium graminearum*, wheat and maize samples

**Fig. 1:**
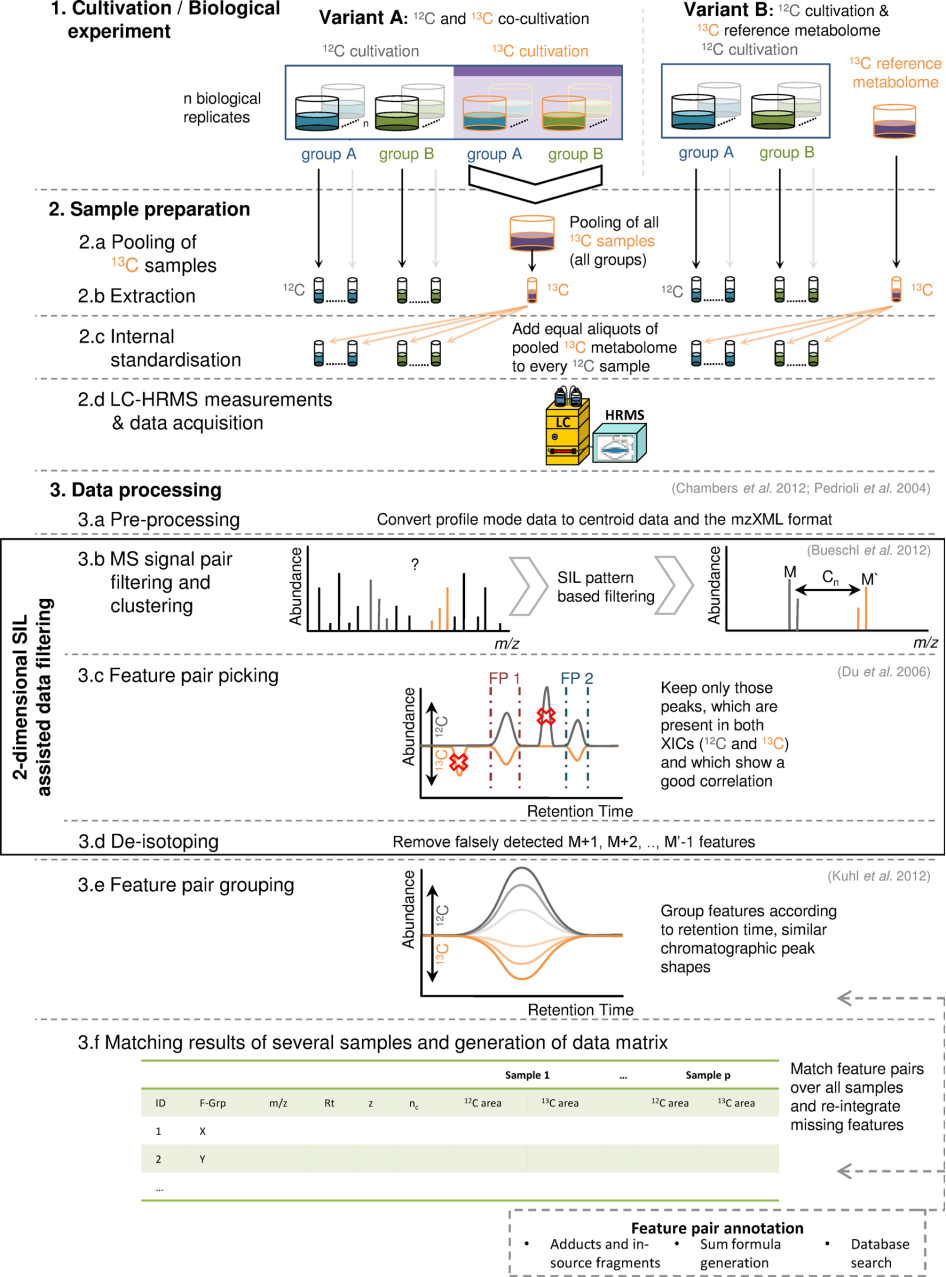
Overview of the proposed SIL assisted workflow for native and U-^13^C co-cultivation (variant A) and native cultivation and use of U-^13^C reference metabolome (variant B) [figure-width: 174 mm]

In this study a metabolomics workflow for two different cultivation variants using stable isotope labelling is presented (Fig. 1). In the first approach (thereafter referred to as variant A) the biological organism of interest is co-cultivated under identical conditions, using either a ^12^C or ^13^C carbon source respectively. This approach is favoured for less complex organisms such as bacteria, yeasts or filamentous fungi which allow cultivation on a defined minimal medium, where the natural carbon source can be easily replaced by highly (>98 %) ^13^C- or ^15^N-enriched nutrients. Variant A is demonstrated for the filamentous fungus *F. graminearum*, a pathogen of several cereal crops. In the second approach (referred to as variant B) a metabolomics experiment is performed using a native ^12^C carbon source for cultivation, while a globally U-^13^C labelled biological sample serves as reference metabolome for IS of the non-labelled experimental samples. This approach is preferred when isotope labelling under experimental conditions is difficult to achieve or not feasible, e.g. with animals or plants. Experimental details are given to a degree necessary to fit the purpose of this paper, which is to present and discuss the analytical concept and data processing rather than the whole biological study.

#### Variant A: ^12^C and ^13^C co-cultivation (*F. graminearum* samples)

*Fusarium graminearum* wild-type (PH-1, NRRL 31084) and its isogenic *tri5*Δ::*loxP* mutant lacking the gene encoding trichodiene synthase, the first enzyme in the trichothecene biosynthetic pathway were used. The strains were cultivated in a modified Fusarium minimal medium (FMM: 1 g/L KH_2_PO_4_, 0.5 g/L MgSO_4_·7H_2_O, 0.5 g/L KCl, 2 g/L NaNO_3_, 10 mg/L citric acid, 10 mg/L ZnSO_4_·6H_2_O, 2 mg/L Fe(NH_4_)_2_(SO_4_)_2_·6H_2_O, 0.5 mg/L CuSO_4_·5H_2_O, 0.1 mg/L MnSO_4_, 0.1 mg/L H_3_BO_3_, 0.1 mg/L Na_2_MoO_4_·2H_2_O) (Leslie and Summerell 2007) containing either non-labelled glucose or U-^13^C-glucose as sole carbon source at a concentration of 10 g/L. *F. graminearum* wild-type and *tri5*Δ strains were grown on non-labelled as well as on U-^13^C labelled FMM using six biological replicates per strain and nutrition condition resulting in a total of 24 samples. The cultures were set up as follows: the strains were sporulated in mung-bean medium as described before by Kluger et al. (2013). 1-ml aliquots of either non-labelled or U-^13^C labelled glucose containing medium were pipetted to each well of a UNIFILTER 24-well 10 ml filtration microplate equipped with a Whatman GF/C filter (VWR, Vienna, Austria) and each well was inoculated with 2,000 spores of the respective *F. graminearum* strain. Still cultures were grown at 20 °C in the dark for 7 days.

#### Variant B: ^12^C cultivation & ^13^C reference metabolome (wheat and maize samples)

Seeds of the wheat cultivars “Remus” and “CM-82036” were grown in pots with soil under environmentally controlled greenhouse conditions. Light and watering regime, humidity and temperature were kept under controlled conditions whenever possible and readjusted continuously to fit the plant’s actual developmental stage. At the onset of anthesis five ears (ten spikelets per ear) were harvested for each cultivar at the beginning of a luminescence cycle and immediately shock-frozen in liquid nitrogen to quench cellular metabolism. In total ten ears were sampled and stored at −80 °C until further sample preparation.

Seeds of the maize line CO354 were planted in pots and later transferred to an environmentally controlled greenhouse with controlled light regime and temperature conditions. Ears were harvested 18 days after hand pollination and kernels were immediately extracted using sterilised scalpels. Three pools of kernels were obtained, where each pool derived from the mixing of seeds came from three different ears, samples were immediately frozen in liquid nitrogen after collecting and stored at −80 °C for further analyses.

For IS using a U-^13^C labelled reference metabolome according to variant B (Fig. 1), a U-^13^C labelled wheat ear (>97 % ^13^C, cultivar Baldus), and U-^13^C labelled maize kernels (>97 % ^13^C, cultivar Yukon chief) were used respectively. It was taken into consideration that the labelled plant material had been grown to the same development stage as the non-labelled wheat cultivars and the native maize line CO354 respectively.

### Sample preparation

#### Variant A: preparation of *F. graminearum* samples

The 24-well microtiter plate was removed from the climate chamber and immediately centrifuged for 10 min at 2,000 rpm to separate the mycelium from the extracellular metabolites in the supernatant. Non-labelled and U-^13^C labelled supernatants were prepared in parallel according to the following protocol. 500 μl aliquots of supernatants of wildtype and *tri5*Δ mutant, which had been grown on native glucose containing FMM were transferred separately each into a 1.5 ml Eppendorf tube resulting in a total of 12 samples (*n* = 6 replicates per fungal strain). 400 μl aliquots of each of U-^13^C labelled supernatants were pooled together in a 50 ml polystyrene tube (VWR International GmbH, Vienna, Austria) resulting in a total of 4,800 μl of a pooled U-^13^C-supernatant for IS. Immediately after centrifugation and aliquoting of supernatants, all aliquots were quenched with 30 % acetonitrile (v/v) resulting in a 7:3 (v/v) ratio of supernatant to acetonitrile. For LC–HRMS analysis, each quenched non-labelled supernatant was standardised by adding the same volume (200 μl) of the pooled and quenched ^13^C-supernatant resulting in LC–HRMS sample aliquots (1:1, v/v).

In addition, 60 μl of each of the U-^13^C-standardised LC–HRMS samples (*n* = 6 replicates per fungal strain) were merged to an aggregate sample (AG) which was used to evaluate the precision of the LC–HRMS measurement and chromatographic peak integration steps by repeated injection out of the same HPLC vial (*n* = 13 replicates). LC–HRMS analysis of all samples was carried out immediately after sample preparation.

For comparison of SIL assisted data processing and conventional data processing by XCMS as well as to demonstrate the extraction efficiency, aliquots of quenched native supernatants without any U-^13^C labelled material were mixed with quenched FMM (1:1, v/v) to yield LC–HRMS samples exhibiting same concentration levels of non-labelled metabolites as the U-^13^C labelled standardised analogues.

To further exemplify the selectivity of the proposed workflow to identify only SIL derived biological information, solvent blanks (water:acetonitrile (7:3, v/v)) containing purified water instead of supernatant were prepared in parallel according to the same procedure mentioned above.

#### Variant B: preparation of wheat and maize samples

The sample preparation of plant material was based on De Vos et al. (2007) carried out after slight modifications as reported in Kluger et al. (2012). Native wheat ears and U-^13^C labelled wheat ear “Baldus” were extracted and prepared in parallel. Native wheat ears of the cultivar “Remus” and “CM-82036” respectively (*n* = 5 replicates per cultivar) were milled separately to a fine powder using a ball mill (MM301 Retsch, Haan, Germany). 100 ± 5 mg of homogenised plant material were weighed to 1.5 mL-Eppendorf tubes with subsequent extraction using 1 mL of pre-cooled (4 °C) methanol:water (3:1, v/v) including 0.1 % formic acid (v/v) in an ultrasonic bath. After centrifugation an aliquot of the supernatants (300 μl of native samples and 420 μl of U-^13^C labelled reference sample) were transferred separately to another 1.5 ml-Eppendorf tube and pre-cooled (4 °C) water + 0.1 % formic acid (v/v) was added to achieve a final methanol:water ratio of 1:1 (v/v). IS was achieved by adding the same volume of U-^13^C labelled sample aliquots resulting in (1:1, v/v) mixtures of non-labelled and U-^13^C labelled supernatant. All samples were rigorously mixed for 10 s before transfer into HPLC vials for LC–HRMS measurements.

Maize line CO354 (*n* = 3) and U-^13^C labelled cultivar “Yukon chief” were prepared according to the same protocol, with “Yukon chief” diluted sample extracts being used for IS.

### LC–HRMS analysis

All samples (*F. graminearum,* wheat, maize) were analysed on a UHPLC system (Accela, Thermo Fisher Scientific, San Jose, CA, USA) coupled to an LTQ Orbitrap XL (Thermo Fisher Scientific) equipped with an ESI source. A HTC PAL system (CTC analytics, Zwingen, Switzerland) was used for injection (10 μl) per sample and for thermostatisation of sample solutions to 10 °C throughout the whole sequence.

A reversed-phase XBridge C_18_, 150 × 2.1 mm i.d., 3.5 μm particle size (Waters, Milford, MA, USA) analytical column, preceded by a C_18_ 4 × 3 mm i.d. security cartridge (Phenomenex, Torrance, CA, USA) was thermostated to 25 °C and used for chromatographic separation at a constant flow rate of 250 μl/min. Water containing 0.1 % FA (v/v) (eluent A) and MeOH containing 0.1 % FA (v/v) (eluent B) were used for linear gradient elution: The initial mobile phase composition (10 % eluent B) was held constant for 2 min, followed by a linear gradient to 100 % eluent B within 30 min. After a hold time of 5 min the column was re-equilibrated for 8 min at 10 % eluent B. A 10 μl sample loop was employed to maintain a constant injection volume.

The ESI interface was operated in positive ion mode with the following settings: sheath gas: 60 arbitrary units, auxillary gas: 15 arbitrary units, sweep gas: 5 arbitrary units, capillary voltage: 4 kV, capillary temperature: 300 °C. LTQ parameters were automatically tuned for maximum signal intensity of a 10 mg L^−1^ reserpine solution (Sigma Aldrich) as recommended by the instrument manufacturer. For measurements using the FT-Orbitrap in the fullscan mode, the automatic gain control was set to a target value of 3 × 10^5^ and a maximum injection time of 500 ms was chosen. The mass spectrometer was operated in a scan range from *m/z* 100–1,000 with a resolving power setting of 60,000 FWHM (at *m/z* 400). Data were recorded using Xcalibur 2.1.0 (Thermo Fisher Scientific).

### Data processing

**Fig. 2:**
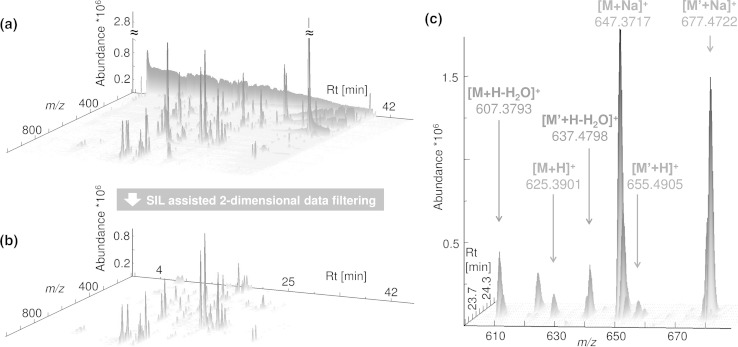
3D representation of a selected *F. graminearum* aggregate sample analysed with LC–HRMS. Chromatogram of the unprocessed, centroided (**a**) and the processed (**b**) with only the SIL derived MS signals are shown. The 3D representation in c shows a zoomed section of the unprocessed datafile (**a**) illustrating the labelling specific isotopic pattern for three different ion species (*M* denotes the monoisotopic ^12^C metabolite and *M*′ denotes the U-^13^C labelled metabolite) of a metabolite with the neutral, monoisotopic mass of 624.3827 u and *n*_C_ = 30 carbon atoms. 3D representations were created with TOPPView (Sturm and Kohlbacher 2009, v. 1.10) [figure-width: 174 mm]

**Fig. 3:**
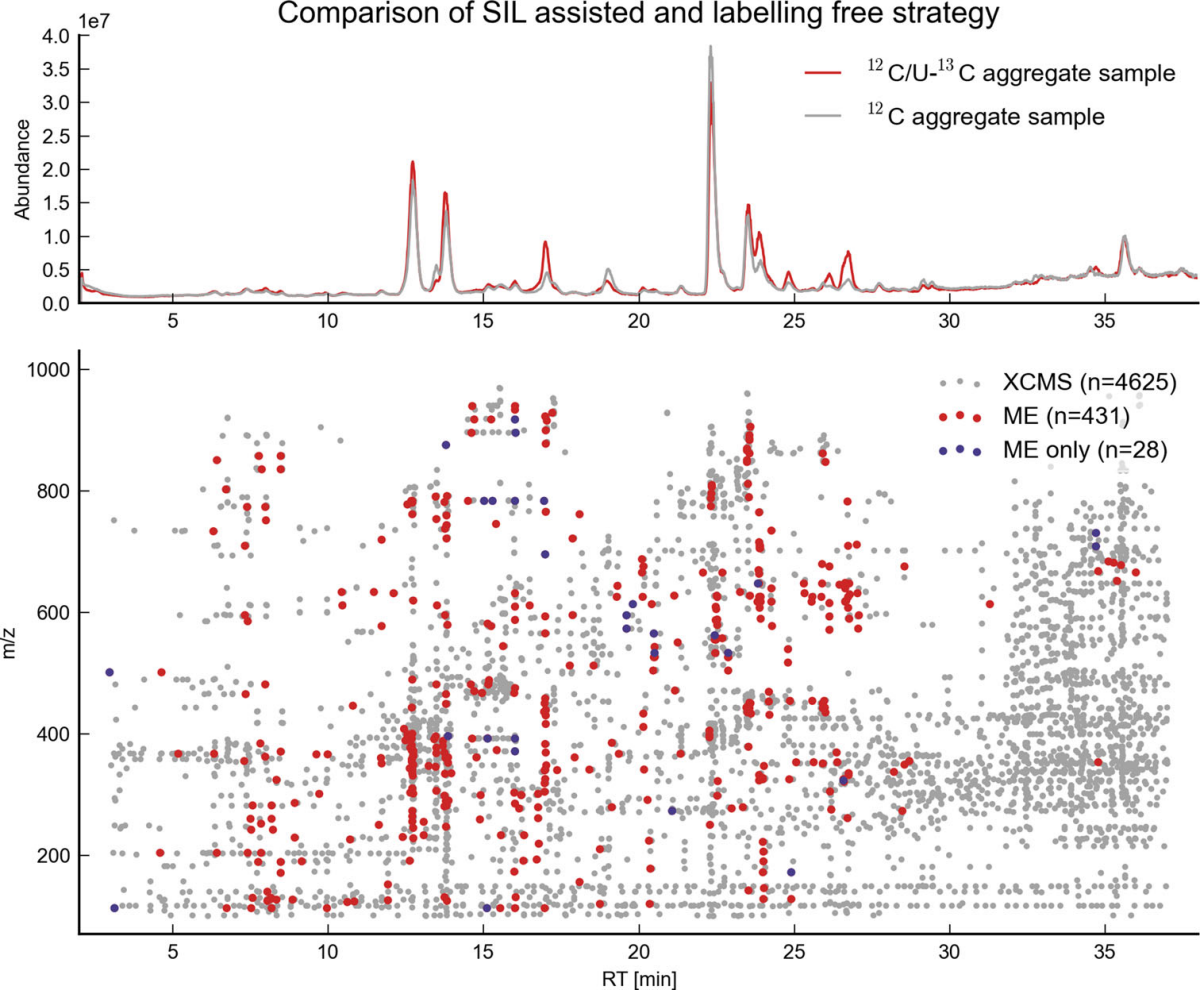
**a** Illustration of an overlay of full scan LC–HRMS total ion current chromatograms obtained for two *F. graminearum* aggregate samples. *Red* Non-labelled ^12^C and U-^13^C culture filtrate mixed 1:1 (v/v); *grey* Non-labelled filtrate mixed 1:1 with fungal growth medium. **b** 2D plot of detected LC–HRMS features (*all dots*). *Grey symbols* indicate all features found with XCMS processing. *Red symbols* represent monoisotopic ^12^C features found by both XCMS and the presented workflow (*variant A*, Fig. 1). Monoisotopic ^12^C features found by the labelling assisted approach only are marked in *blue*. Features with a retention time >30 min are mainly detected by XCMS. Due to the higher strength of the eluent, predominantly impurities of non-biological origin such as polymers and apolar compounds are displaced from the stationary phase [figure-width: 174 mm]

For an efficient extraction of metabolite derived MS signals and analytical features, several consecutive data processing steps (illustrated in Fig. 1-3-a–f) were implemented as an expansion of the already released software version of MetExtract (Bueschl et al. 2012). The focus of this paper was laid on the detailed description of the complete workflow including the biological experiment, sample analysis and data processing as well as its application to samples of plants and fungi. The extended MetExtract 2.0 software, which is capable of performing all of the following data processing steps (2.5.1–2.5.7) will be published elsewhere together with the release of the programme.

#### Pre-processing (Fig. 1-3-a)

Measurement files were converted from acquired profile to centroid data and the mzXML format (Pedrioli et al. 2004) with the MSConvert programme from the freely available ProteoWizard package v.3.0.3980 32-bit (Chambers et al. 2012).

#### MS signal pair filtering and clustering of m/z values (Fig. 1-3-b)

Each recorded MS scan was inspected for the typical SIL derived isotopic pattern as described previously in Bueschl et al. (2012). The intensity threshold of both the monoisotopic ^12^C derived (M) and U-^13^C derived (M′) MS signal was set to 5,000 counts in at least 3 scans. The maximum *m/z* deviation from postulated *m/z* values was set to 2.5 ppm and the isotopologue abundance error was set to ±20 %. Each MS signal pair, fulfilling the criteria was annotated with *m/z* of M, the charge number z, deduced from the SIL derived isotopic pattern, and the determined number of carbon atoms n_C_, calculated from the *m/z* value difference between M and M′, and z. Extracted MS signal pairs were clustered together with hierarchical clustering to group redundantly extracted MS signal pairs of similar *m/z* value (i.e. MS signal pairs, which originate from the same chromatographic peak or structural isomers with identical sum formula). Hierarchical clustering was performed separately for all MS signals having the same number of carbon atoms and the same charge number. All clusters in the resulting tree, whose *m/z* values differed more than ±10 ppm were split into separate sub-clusters.

#### Feature pair picking (Fig. 1-3-c)

For each MS signal cluster, the algorithm of (Du et al. 2006) was utilised to inspect the XICs of both the monoisotopic ^12^C and the corresponding U-^13^C labelled ions for co-eluting and similarly shaped chromatographic peaks. For this purpose, a maximum retention time difference of ±15 scans was tolerated between chromatographic peaks in both XICs. Furthermore, the chromatographic peak profiles of the monoisotopic ^12^C and the U-^13^C labelled features were compared with the Pearson correlation coefficient and only those, with correlation coefficients greater than 0.5 were considered a valid feature pair derived from the SIL process. This data processing step resulted in a list of putative feature pairs (monoisotopic ^12^C- and corresponding U-^13^C labelled feature) with each feature pair being annotated with the *m/z* value of M, retention time (Rt), peak area, number of carbon atoms per ion (n_C_), and charge state z.

#### De-isotoping (Fig. 1-3-d)

Compared to correctly paired features, M+1 features, falsely picked as monoisotopic ^12^C features or M′−1 features, falsely picked as U-^13^C labelled features showed a reduced number of carbon atoms n_C_ and/or an increased monoisotopic ^12^C *m/z* value. Thus such erroneously extracted features were removed from the feature list by comparing the *m/z* values of M, charge state z, Rt and n_C_ among putative feature pairs.

#### Feature pair grouping (Fig. 1-3-e)

To group different features from the same metabolite, extracted feature pairs were convoluted by comparing the chromatographic peak shapes of all monoisotopic ^12^C features eluting at approximately the same retention time (±10 scans) (Kuhl et al. 2012). A minimum correlation coefficient of 0.85 was specified for features to be grouped together.

#### Matching results of several samples and generation of data matrix (Fig. 1-3-f)

To track metabolite features over all samples of a particular experiment, the extracted feature pairs of all LC–HRMS data files were compared using n_C_, *m/z* of M and Rt in that order. After data matrix generation, monoisotopic ^12^C and U-^13^C labelled features, initially missed in some of the data files due to the restrictive filtering criteria, were searched for in a targeted way. To this end, the described peak picking and integration algorithms were employed but without checking peak shape similarity.

#### Internal standardisation

Internal standardisation was carried out on a file basis for each feature pair by dividing the area of monoisotopic ^12^C by that of its corresponding U-^13^C labelled feature.

### Comparison with labelling free strategy

To compare the feature extraction process with a labelling free approach, a non-labelled *F. graminearum* aggregate sample, which had been diluted with FMM (1:1, v/v) and did not contain any U-^13^C labelled culture supernatant and one of the U-^13^C standardised *F. graminearum* aggregate samples, were analysed, processed and evaluated. The data file derived from a non-labelled sample was processed with XCMS (1.34.0) and R (R Development Core Team, 2012, v. 2.15.2) using parameter settings as recommended by Patti et al. (2012a) for HPLC Orbitrap XL MS. The LC–HRMS data file obtained for the U-^13^C standardised aggregate sample was processed as described above (steps 2.5.1–2.5.4) and parameter settings similar to XCMS (i.e. minimum intensity of 5,000 in at least 3 scans; maximum tolerated *m/z* deviation of 2.5 ppm). Automated comparison of the results was performed by comparing both the determined *m/z* value and retention time of all extracted features and feature pairs respectively. For this, a maximum relative *m/z* deviation of ±10 ppm and ±0.15 min was allowed for two results to match. Features, which had only been found by the SIL assisted data processing, were further inspected manually using TOPPView (Sturm and Kohlbacher 2009, v 1.10).

### Selectivity evaluation of SIL assisted workflow

To demonstrate the selectivity of feature pair extraction in the presented SIL assisted metabolomics workflow, blank samples (solvent blank) (*n* = 3) as well as five non-labelled *F. graminearum* aggregate samples (no internal standardisation with U-^13^C labelled supernatant) were processed as described earlier.

### Evaluation of internal standardisation and matrix effects

Analysis of internal standardisation was performed on a feature pair level using only those pairs for which both the monoisotopic ^12^C and the corresponding U-^13^C labelled features were found in all replicates of a certain sample type after re-integration. Therefore, no imputation of missing values was required. For analytical precision demonstration before and after internal standardisation coefficient of variance (CV) histograms of individual feature pairs within all replicates of a sample type were calculated. The bin width was set to 5 %. CV values above 120 % were set to 120 % to achieve equidistant axis in the plots. To demonstrate internal standardisation with multivariate statistics, PCA plots were calculated for the monoisotopic ^12^C and U-^13^C labelled feature areas as well as for the internal standardisation derived feature pairs. For analytical precision analyses R (R Development Core Team 2012 v. 2.15.2) was used. The functionality for calculating the principal component analysis (PCA) was taken from the package ChemometricsWithR (Wehrens 2011, pp. 53–57). Data were range scaled (van den Berg et al. 2006) prior to PCA. For the ellipsis in the PCA plots, the ellipse package (Murdoch and Chow 1996) was used. Ellipses were calculated using the co-variance matrices of PC1 and PC2 of the respective sample types.

## Results and discussion

U-^13^C or ^15^N labelled metabolites show nearly identical physico-chemical properties as their native non-labelled analogues. As a consequence, LC–HRMS measurements of mixtures of non-labelled and U-^13^C labelled biological samples result in perfect co-elution of all isotopologues of a particular metabolite with very similar chromatographic peak shapes (Fig. 2). Thus, the analysis of mixtures of native and U-^13^C labelled biological samples leads to labelling-specific isotopic distributions of both the non-labelled and U-^13^C labelled metabolites in all recorded mass spectra containing biologically derived ion signals. As can be expected from the ESI process, different ion species such as protonated molecules as well as sodium adducts or the loss of water from the intact molecules may be observed. For each of the detected ion species two distinct mirror-imaged isotopic patterns are present in the mass spectra. In addition to the regular signal pattern originating from the natural isotopic composition of carbon (98.8 % ^12^C and 1.1 % ^13^C), the second isotopic pattern shows ascending MS signal intensities towards higher *m/z* values for all U-^13^C labelled metabolite derived ion species. The relative abundance of the isotopic signals in the pattern of the labelled metabolite is given by the degree of ^13^C enrichment achieved in the respective experiment (variant A) or the labelled biological reference sample (variant B). For the cultivation of *F. graminearum* strains (variant A) the degree of ^13^C enrichment of metabolites was estimated from one data file using highly abundant features of both the monoisotopic ^12^C and the corresponding U-^13^C labelled isotopologues. From the intensity ratio of M′−1 to M′ as well as the deduced number of carbon atoms for this isotopic pattern, the enrichment was calculated to be as high as 99.5 %, which is in good agreement with the suppliers specifications (99 %). For the wheat and maize samples (variant B), the U-^13^C labelled reference samples also corresponded well to the supplier’s specifications of around 97.5 %.

Variant A can be realized without much extra effort for less complex organisms such as bacteria, yeasts or fungi, which can be grown on synthetic media that require only a limited number of carbon sources, available as stable isotope enriched nutrients. Growing U-^13^C labelled plants (e.g. *A. thaliana*, wheat) has also been carried out successfully but is a more challenging task regarding infrastructure, costs, and time to establish cultivation conditions in a controlled U-^13^CO_2_ enriched atmosphere and can be demanding, particularly under the experimental conditions of interest. If U-^13^C labelled plant material is commercially available, variant B might be a good compromise as it provides the possibility to use globally U-^13^C labelled plant samples as reference for both qualitative and quantitative measurements with the drawback however, that the biological experiment itself is not carried out under labelling conditions. Mammalian organisms can be regarded to be even more demanding than plants. So far, successful SIL experiments (^15^N or ^13^C stable isotope enrichment >90 %) of different mammalian cell lines (e.g. CHO) requiring complex media have been reported (Egorova-Zachernyuk et al. 2011), but to the best of our knowledge, labelling of whole mammalian organisms was not performed successfully to date.

With the presented approach, the systematic search of MS-signals and feature pairs carrying the SIL specific isotopic pattern virtually enables the complete annotation of that part of the metabolome of a biological sample which can be accessed by the chosen sample preparation and measurement method. Moreover, since the mass difference of the ^12^C monoisotopic ion M and its U-^13^C labelled isotopologue ion M′ is proportional to the mass difference between ^12^C and ^13^C isotopes (i.e. 1.00335 u), the number of carbon atoms contained in that particular metabolite ions can directly be calculated from the measured HRMS spectra (Bueschl et al. 2012). A prerequisite to unambiguously assign the number of carbon atoms (n_C_) in a particular metabolite ion directly, the workflow requires the *m/z* value of both the monoisotopic, non-labelled M- and fully ^13^C labelled M′ ions to be clearly identifiable among their respective isotopic patterns (Fig. 2c). For example, a ^13^C enrichment of 98 % for the labelled metabolites allows extracting and directly calculating the number of carbon atoms in metabolite ions containing up to 60 carbon atoms. In case the degree of ^13^C enrichment dropped to e.g. 85 %, this number is reduced to a maximum of 7 carbon atoms before the MS signal of a hypothetical ^13^C _n-1_^12^C_1_ reached the same intensity as the fully labelled ^13^C_n_ isotopologue and thus would interfere with direct n_C_ assignment. It should be noted however that even at a further reduced enrichment degree of as low as e.g. 75 or 50 %, the presented workflow does still allow for the automated recognition of corresponding isotopic ion patterns when parameter settings for processing are adjusted accordingly. In addition to the qualitative aspects of improved metabolome annotation, the use of globally U-^13^C labelled biological samples permits a highly efficient internal standardisation thereby enabling the assessment of precision parameters (both biological and technical) as well as compensation of matrix effects and improved relative quantification of hundreds of metabolites simultaneously.

### Feature reduction by two-dimensional data filtering and feature grouping

With an average number of approximately 2 million signals (corresponding to 900 MS signals/mass scan), the raw chromatograms of *Fusarium* culture supernatants contained less data points than the plant derived chromatograms carrying roughly 3 million MS signals (1,200 MS signals/mass scan). This greater complexity of the plant samples is also visible in all successive data processing steps. The first data filtering step, comprises an inspection in every mass spectrum of a particular data file for groups of corresponding M (i.e. monoisotopic ^12^C metabolite ion), M+1, M′ (i.e. U-^13^C labelled metabolite ion) and M‘−1 isotopologue MS signals forming MS signal pairs. The formation of MS signal pairs in each mass scan resulted in the most significant reduction of data points. As illustrated for a selected *F. graminearum* aggregate sample (respective numbers for other sample types and experiments can be found in Table 1), the LC–HRMS raw chromatogram (centroided and converted to mzXML) contained a total of 1,987,654 MS signals which were reduced by a factor of about 120 to 16,736 putative M/M′ signal pairs under the tested conditions (Fig. 2a, b). On average, the number of extracted signal pairs represents 0.6–0.9 % of the original contained MS signals. It should be noted that low abundant principal ions (i.e. M or M′) may not show distinctive M+1 or M′−1 isotopic signals (e.g. at the beginning/end of a chromatographic peak), and thus these signals are not considered during this filtering step. Therefore, the number of real SIL derived signal pairs is always underestimated. However, data processing which does not verify the isotopic patterns could successfully extract these low abundant metabolite ions, but at the same time increase the number of false positive findings (e.g. pairings of artefacts which can originate from the Fourier transformation process (Brown et al. 2009)). However, all SIL assisted data processing steps presented here always verify the isotopic patterns of both the native and U-^13^C labelled metabolite ions using the number of carbon atoms for this ion species deduced from M and M′ respectively.

**Table 1:** The table provides a quantitative overview of the data processing results with the proposed workflow (Fig. 1)

No.	Workflow step	Wildtype PH-1 (variant A)	Aggregate samples (variant A)	Remus wheat (variant B)	CM wheat (variant B)	CO354 maize (variant B)
MS signals	3-a	1.8 × 10^6^ (±0.06 × 10^6^)	1.9 × 10^6^ (±0.09 × 10^6^)	3.0 × 10^6^ (±0.01 × 10^6^)	3.0 × 10^6^ (±2 × 10^2^)	2.8 × 10^6^ (±7 × 10^3^)
SIL derived signal pairs	3-b	1.06 × 10^4^ (±0.24 × 10^4^)	1.65 × 10^4^ (±0.52 × 10^4^)	1.87 × 10^4^ (±0.66 × 10^4^)	2.09 × 10^4^ (±0.19 × 10^4^)	2.04 × 10^4^ (±0.67 × 10^4^)
MS signal clusters	3-b	8.09 × 10^2^ (±0.27 × 10^2^)	1.28 × 10^3^ (±0.04 × 10^3^)	2.07 × 10^3^ (±0.03 × 10^3^)	2.32 × 10^3^ (±0.13 × 10^3^)	1.39 × 10^3^ (±0.37 × 10^3^)
Feature pairs before de-isotoping	3-c	824 (±19)	1,199 (±34)	1,288 (±30)	1,443 (±92)	858 (±274)
De-isotoped feature pairs	3-d	291 (±9)	442 (±14)	797 (±9)	902 (±68)	511 (±166)
Feature groups (i.e. metabolites)	3-e	87 (±6)	135 (±6)	347 (±12)	362 (±32)	209 (±58)

Selected sample types are taken from the *F. graminearum*, wheat and maize experiment. The mean value and its standard deviation among the replicates within a certain sample type are given Table 1

After MS signal clustering, feature pairs are extracted from the data. As the non-labelled monoisotopic and its corresponding U-^13^C labelled analogue of a particular metabolite can be expected to show perfect chromatographic co-elution, verification of retention time and chromatographic peak shape similarity is used for feature pair picking (Fig. 1-3-c). The subsequent de-isotoping step eliminates incorrectly paired monoisotopic ^12^C- and U-^13^C features which do not represent true monoisotopic or uniformly labelled features. Together, the data filtering steps 3b–3d reduced the metabolite-related information—depending on the investigated organism—to ca. 300–900 distinct de-isotoped feature pairs per LC–HRMS chromatogram.

Since ionisation by electrospray may give rise to several ion species for the same substance such as adducts, in-source fragments or dimers, the SIL derived feature pairs are further combined with the aim to convolute all ion species of a particular metabolite into single groups (Fig. 1-3-e). Feature grouping is greatly facilitated by the prior removal of all non-biology related as well as all M+1, M+2 isotopic features and, depending on the investigated samples, resulted in the detection of 87–135 metabolites for the *F. graminearum*—and 200–360 truly plant derived substances for the maize and the wheat extracts respectively. As a major benefit of the presented approach all of these metabolites can be used to build-up reference databases and can serve as positive lists for future metabolomics experiments. The list of approximately 135 metabolites detected for the *Fusarium* aggregate samples (variant A, labelling under experimental conditions) can be assumed to comprise all metabolites which have been produced and released by at least one of the fungal strains under the tested conditions. For variant B of the workflow, which is exemplified with globally U-^13^C-labelled reference samples, the detected metabolites are restricted to those present in both, the reference as well as the experimental samples. Nevertheless, all of the generated feature groups facilitate metabolic feature annotation and come with additional valuable characteristics for molecular formula generation and metabolite annotation such as the number of carbon atoms, the charge state for each metabolite ion as well as all other ion species detected for that particular metabolite (i.e. feature group).

#### Comparison of feature extraction with a labelling-free workflow

To compare the number of SIL derived features with those found with a labelling-free metabolomics approach, a selected native *F. graminearum* aggregate sample (consisting of a mixture of non-labelled *F. graminearum* wildtype and non-labelled *tri5*Δ mutant culture supernatants) was diluted 1:1 (v/v) with either *Fusarium* minimal medium (FMM) or a pooled ^13^C-aggregate supernatant, to generate two LC–HRMS samples both containing the native metabolites at identical concentration levels. Retention time shifts observed between the respective LC–HRMS chromatograms were negligible (see TICs in Fig. 3a). Parameter settings for XCMS-based (Smith et al. 2006) feature extraction for the non-labelled aggregate sample chromatogram such as intensity threshold (5,000 counts), minimum number of scans (3×), maximum ppm deviation (2.5 ppm) were kept identical to the SIL assisted feature picking in order to ensure maximum comparability of results between the two approaches. As expected, with XCMS every chromatographic peak in the data regardless of its origin (biological, background, noise…) was extracted as a feature. In total, *n* = 4,625 features (illustrated as grey symbols in scatter plot of Fig. 3b) were found by XCMS based data processing including all low abundant features with no observable isotopic peaks or MS signals. In contrast, with the SIL assisted approach MS signal- and feature pair picking and subsequent de-isotoping only yields monoisotopic ^12^C features with a high degree of confidence to correspond to truly sample derived metabolites. In total, application of the SIL assisted workflow yielded 431 feature pairs (red dots in scatter plot in Fig. 3b) which are about ten times less compared to XCMS. Moreover, data processing and automated comparison of the SIL and XCMS assisted approach resulted in 28 feature pairs (Fig. 3b blue dots) solely found with the SIL assisted approach. A closer, manual inspection of these 28 results showed, that 22 features were not automatically matched because of larger *m/z* or retention time deviations. Only three feature pairs found solely with the SIL assisted approach did not show any signals in the native sample processed with XCMS. Moreover, 3 of these 28 feature pairs were identified as false positives (e.g. pairings of Fourier transform artefacts (Brown et al. 2009)). For additional manual inspection of those parts of the LC–HRMS chromatograms showing a high density of features (e.g. Rt ≥ 30 min or *m/z* ≤ 150), which had only been found by XCMS, TOPPView was used to confirm that none of these features did show corresponding U-^13^C labelled isotopologues.

It should be noted that workflows which do not make use of SIL, generally try to not further consider those non-biologically related background features by using statistical analysis to select features significantly differing between two or more experimental conditions. Such an assumption implies, however that only biologically derived features vary significantly between the different experimental conditions and that all others approximately show similar abundances across the different sample types.

#### Selectivity of the presented approach

To demonstrate the selectivity of the workflow for the extraction of truly biologically related metabolite features by the use of SIL, eight blank samples were included in a measurement sequence of *F. graminearum* samples and evaluated according to the presented workflow (variant A). Three of these blanks, which consisted of purified water instead of fungal supernatant, were employed as solvent blanks. Furthermore, five aggregate samples containing only the non-labelled *F. graminearum* metabolome served as simulated matrix background blanks. Metabolite ions detected in the native *F. graminearum* supernatants were not expected to be found with the SIL assisted data processing steps since they did not contain any U-^13^C labelled metabolites. In the solvent blanks, hardly any MS signal pairs (searched in each mass scan, Fig. 1-3-b) were extracted (2, 26 and 39 MS signal pairs respectively). Better yet, none of these MS signal pairs were further confirmed to be valid feature pairs according to the predefined filtering criteria (Fig. 1-3-c). The simulated background blanks showed 14, 17, 7, 224 and 106 MS signal pairs on a mass scan level. Subsequent feature pair picking revealed 0, 1, 1, 1 and 5 feature pairs for the simulated background blanks respectively. However, none of such extracted feature pairs were found in more than one of the measured matrix blanks. Further manual inspection clearly showed that all of these randomly picked feature pairs fulfilled the present criteria either by chance or were pairings of different adducts or Fourier transformation artefacts (Brown et al. 2009). Such incorrectly picked adducts or artefacts showed nearly identical chromatographic profiles and they were therefore not discarded as false positives automatically (Fig. 1-3-e). Two of these feature pairs were detected at >700 u with a difference between the monoisotopic ^12^C and corresponding U-^13^C mass corresponding to less than ten carbon atoms. Thus such feature pairs can easily be excluded from further data analysis. In conclusion the very low rate of false positives in both types of blank samples demonstrates the exceptionally high selectivity of the presented approach in only extracting truly biologically related feature pairs.

### Results of internal standardisation

Absolute quantification in untargeted metabolomics experiments of all detected (known or unknown) metabolites is generally not feasible by most of the current approaches. Moreover, the accuracy of relative feature/metabolite quantification in untargeted metabolomics experiments is limited by matrix effects which can cause problems during statistical analysis as the biased feature abundances complicate comparison across different experimental conditions. Internal standardisation using globally stable isotope labelled biological samples provides the ideal tool to overcome these limitations and has already been used for relative (e.g. Giavalisco et al. 2009) and even for absolute quantification in untargeted metabolomics approaches (Bennett et al. 2008). As the presented workflow also makes use of globally U-^13^C labelled biological samples, the effect of global internal standardisation on matrix effects and technical precision has been investigated at the example of the *F. graminearum* dataset.

#### Correction of matrix effects

**Fig. 4:**
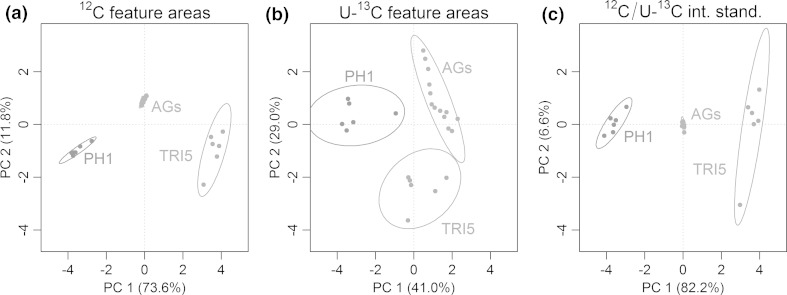
Three PCA *scores plots* derived from consistently extracted feature pairs of three sample types: *F. graminearum* samples PH-1, *tri5*Δ and aggregate samples (AGs). For all three PCAs the exactly same set of feature pairs was used, however different intensity values (*peak areas*) were taken for each feature pair. **a** areas of monoisotopic ^12^C features of the respective feature pairs, **b** areas of U-^13^C labelled features, **c** intensity ratios of monoisotopic ^12^C and corresponding U-^13^C feature area (internal standardisation) [figure-width: 174 mm]

In many metabolomics experiments unsupervised multivariate statistical tools such as PCA are used as a first step to reduce the dimensionality of the analytical data and test for separation of biological samples into different classes according to experimental conditions. Such tools operate on different signal abundances or feature areas in the data matrix obtained from prior data processing. In order to test the suitability of the SIL assisted workflow to correct for matrix effects, feature areas obtained for the *F. graminearum* dataset were range scaled (van den Berg et al. 2006) and subsequently PCA score plots were calculated using (1) peak areas of monoisotopic ^12^C features (^12^C-PCA, Fig. 4a), (2) U-^13^C labelled ion species (U-^13^C-PCA, Fig. 4b) and (3) the peak area ratio of the respective non-labelled and U-^13^C features of a certain feature pair (^12^C/U-^13^C-PCA, Fig. 4c). To be able to compare the three different PCA plots, only those feature pairs which had been found consistently throughout all replicates and samples categories (PH-1, *tri5*Δ and pooled aggregate samples (AGs)) were considered (*n* = 109).

As can be expected from the experiment, the score plot of the ^12^C-PCA shows a clear separation of the PH-1, *tri5*Δ and AGs samples into three distinct groups. The same holds true for the ^12^C/U-^13^C-PCA, which was calculated from the area ratios of non-labelled and corresponding U-^13^C features. Additionally the variance captured by PC1 and PC2 increased slightly from 85.4 to 88.8 %. Compared to the ^12^C-PCA, the aggregate samples are located in the centre of the ^12^C/U-^13^C-PCA score plot (Fig. 4c), which is explained by the range scaling process together with the fact that these aggregate samples constitute of equal amounts of U-^13^C internally standardised PH-1 and *tri5*Δ samples. In contrast to the ideal behaviour of the U-^13^C-standardised AG samples, matrix effects obviously affected the monoisotopic ^12^C feature areas of the aggregate samples (Fig. 4a), which in turn become visible as a shift of the aggregate sample group away from the centre of the ^12^C-PCA score plot.

Interestingly, the use of U-^13^C-feature areas for PCA also resulted in a clear separation into the three sample categories, although (according to the preparation protocol for the AGs, see Sect. 2.3.1) the absolute concentration levels of all U-^13^C labelled metabolites were identical in every of the analysed samples and sample type. In this case, the separation of the sample groups in the U-^13^C-PCA plots is explained by the different metabolic composition of the wildtype PH-1, *tri5*Δ and AGs samples with respect to their non-labelled metabolites, which resulted in distinct alterations of the areas derived from U-^13^C labelled features (i.e. matrix effects) for each of the tested sample categories. The separation of the three sample categories based on the peak areas of the respective U-^13^C features would have never been recognised as artefact (caused by matrix effects) without the availability of globally U-^13^C labelled biological samples. In contrast, an observation as depicted in Fig. 4b most probably would have led to the false conclusion that metabolites differing between the experimental samples had caused the separation according to the tested experimental states.

#### Precision of workflow and improvement of technical data variability

**Fig. 5:**
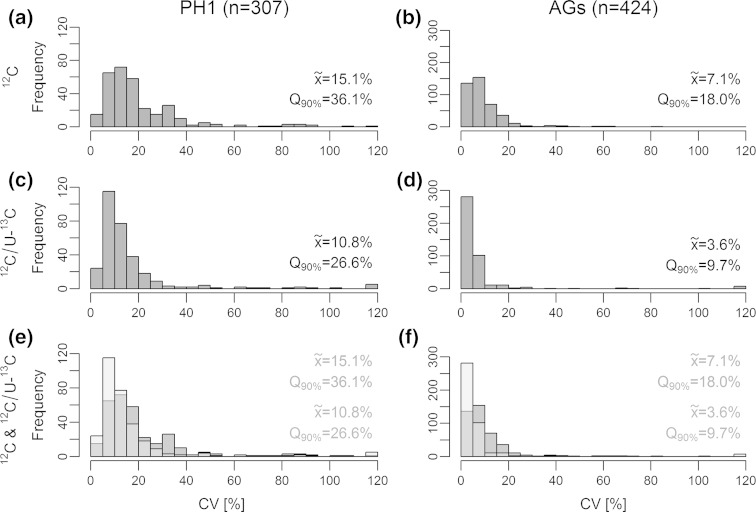
Histograms showing the distributions of coefficients of variation (CV) across all SIL derived features which were consistently found in all replicates of *F. graminearum* wildtype PH-1 (*n* = 6) and *F. graminearum* aggregate samples (*n* = 13). The histograms in **a** and **b** (*red*) were derived from the peak areas of the monoisotopic ^12^C feature of the respective feature pairs while **c** and **d** (*blue*) were calculated after internal standardisation with the areas of the corresponding U-^13^C labelled features of the very same feature pair. Histograms **e** and **f** (overlay of transparent *red* and *blue*) combine the respective above two histograms to illustrate the shift towards lower CVs by internal standardisation, achieved for both sample types [figure-width: 129 mm]

Stable isotope labelling assisted internal standardisation has been successfully used for improved metabolite quantification in GC–MS and LC–MS based metabolomics studies (e.g. (Birkemeyer et al. 2005; Bennett et al. 2008; Giavalisco et al. 2009)). Here, the assessment and improvement of both biological and technical precision of the presented SIL assisted workflow are exemplified with the *F. graminearum* dataset. Again, U-^13^C labelled fungal samples were employed for global internal standardisation of non-labelled samples and subsequently precision measures of the workflow were estimated. Only those feature pairs were considered which had been found in all replicates of the respective sample type corresponding to *n* = 307 ^12^C/U-^13^C feature pairs for PH-1 wildtype data and *n* = 424 for aggregate samples. Coefficients of variation (CVs) of each feature (pair) were calculated across all replicates of a particular sample type, the distribution of CVs was plotted as a histogram with a class size of 5 % (Fig. 5) and the median CV as well as 90 % percentile were taken as precision estimate. For PH-1 samples, CVs of monoisotopic ^12^C feature areas showed a median value of 15.1 % with 90 % of all features showing CVs below 36 %. These CV values can be interpreted as a measure for the variability of the overall workflow including all steps from culturing of fungi (biological variance) to sample preparation, LC–HRMS measurement, data processing and feature integration (technical variance). Using the area ratio of corresponding monoisotopic ^12^C and U-^13^C features, the overall precision of PH-1 samples was improved to a median CV of 10.8 %, with 90 % of all features showing CVs below 26.6 %, indicating that (1) biological and technical variability roughly contributed equally to the overall spread of feature areas and (2) internal standardisation with globally U-^13^C labelled samples helped to improve precision considerably. For the *tri5*Δ mutant, comparable CV values and improvements were obtained (data not shown).

In order to estimate and dissect the precision of the end determination step (i.e. LC–HRMS measurement, data processing and feature integration), ^12^C/U-^13^C aggregate samples, consisting of 1:1 mixtures of PH-1/*tri5*Δ supernatants were measured as replicate injections (*n* = 13) at regular intervals over a complete LC–HRMS sequence. The distribution of CVs of the monoisotopic ^12^C feature areas showed a median CV of 7.1 % (90 % of all features had CV values below 18 %). After internal standardisation (blue histogram), the distribution of CVs shifted left (blue histogram) towards lower CV values with a median and 90 % quartile of to 3.6 and 9.7 % respectively, demonstrating a substantial improvement (~50 %) of the LC–HRMS end determination.

It should be noted that for a few (*n* < 10) features the internal standardisation of *F. graminearum* culture supernatants resulted in CV values >120 %. This might have been caused by non-reproducible degradation/chemical conversion of a few metabolite features in the samples but this phenomenon was not further investigated in this study however.

With ca. 20 % and 40–50 % respectively the distribution of CVs in wheat and maize samples (variant B) yielded slightly higher median and 90 % percentile values than for the *F. graminearum* samples (data not shown). Similar to the *F. graminearum* experiment, wheat and maize aggregate samples were used to study the technical precision. Similar to *Fusarium*, median CV (90 % percentile) values shifted from roughly 8 % (16 %) to 5 % (20 %) for wheat and 12 % (22 %) to 6 % (17 %) for maize respectively (data not shown).

In conclusion, the above illustrated results are in good agreement with the reports of e.g. Birkemeyer et al. (2005), Bennett et al. (2008), Giavalisco et al. (2009), who described enhanced precision and (relative) quantification after metabolome wide internal standardisation by use of U-^13^C labelled biological samples. Furthermore, as shown for *F. graminearum* samples, internal standardisation resulted in an improved performance of multivariate data analysis.

## Concluding remarks

Recent reports of SIL assisted tools and techniques and their application to various fields of metabolomics have illustrated significant improvements to circumvent major challenges of untargeted metabolite profiling. The presented workflow enables the untargeted global extraction of truly metabolite related MS signals and features in LC–HRMS datafiles derived from native and U-^13^C labelled metabolomes. Together with the automated generation of hundreds of feature groups per sample, each of which is representing a distinct metabolite, this approach constitutes a major step forward towards global annotation of the entire metabolic composition of biological samples. Additionally, metabolome wide internal standardisation with U-^13^C labelled samples greatly enhances accuracy and reliability of relative quantification by correction of technical variability as well as correction of matrix effects, which otherwise are difficult to evaluate and compensate.

In conclusion, although stable isotope labelling of whole metabolomes for untargeted metabolomics is still challenging and generally requires additional efforts in terms of costs and/or experimental design, it is anticipated that SIL assisted metabolomics will arouse increasing interest and become a well-established technique in metabolomics research.
